# Supplementary material for: Assessing the knowledge, attitude and practice of osteoporosis among Pakistani women: A national social-media based survey
Source: PLoS One. 2023 Nov 10;18(11):e0288057. doi: 10.1371/journal.pone.0288057 (PMC10637646; doi:10.1371/journal.pone.0288057)
Supplement: Attachment — Submitted filename: Response to Reviewers_18_April.docx [file pone.0288057.s001.docx]

**Response to Reviewers:**

Review Comments to the Author

Please use the space provided to explain your answers to the questions above. You may also include additional comments for the author, including concerns about dual publication, research ethics, or publication ethics. (Please upload your review as an attachment if it exceeds 20,000 characters)

Reviewer #1: 1. It would be better to define primary and secondary outcome variables in methods section in line with objectives. Results, discussion and conclusion need to follow the same in chain.

**Response: Primary and secondary outcomes are now defined in the Methods. The rest of the sections also follow the same in chain.**

2. Methods sections is supposed to be core of any study. Here, methods section contains inadequate information. For example, following components for methods section need to be well described.
i. Details of Study design
ii. Setting
iii. Sample size estimation
iv. Sampling technique
v. Participant
vi. Primary and secondary outcome variables with working definition
vii. Intervention/issue of interest (exposure)
viii. Ethics and end point
ix. Statistical analysis

**Response: The methods are now well defined and in line with the format mentioned above.**

3. The discussion section needs to be described scientifically. Kindly frame it along the following lines:
a. Main findings of the present study
b. Comparison with other studies
c. Implication and explanation of findings
d. Strengths and limitations
e. Conclusion, recommendation and future directions

**Response: Thank you for your feedback. We have modified the section according to your guidance and made changed in the section in the revised manuscript.**

Reviewer #2: This research found that adult Pakistani women have a fair understanding of osteoporosis, but the OPAAT tool clarifies some common misconceptions. There is a need to develop educational strategies to increase the knowledge of osteoporosis among Pakistani adults and to promote a bone-healthy lifestyle. This is an interesting and meaningful study, and I recommend accept.

Reviewer #3: Thank you for the article.
It was a nice read. Obviously, the effort was meant to highlight the issue and find out the misconceptions using validated outcome measure. The authors have just done that. In a survey, there will always be issues with demographics and how was the population selected. Urban Sindh being more tech advanced and educated obviously will sway the percentages in any survey. If the scores could be adjusted according to provinces, this would have been cherry on top.
Best Regards

**Response: Thank you for such a wonderful review, we have tried our best to highlight these disparities in our text, but it was not a focus of our objective hence it is too late to adjust our results now. We have mentioned it in the Limitations section.**

Reviewer #4: Assessing the Knowledge, Attitude and Practice of Osteoporosis among Pakistani
Women: A National Social-Media Based Survey

Review
Thank you for letting me review your article. This study assesses Pakistani women's knowledge, attitudes, practices about osteoporosis by using OPAAT. The content is useful for educating Pakistanis about osteoporosis in the future.

Methods
Line70-71 It is not clear on how you arrived at the sample size, please describe what you estimated the absolute precision and expected response rate to be.

**Response:**

**For sample size, an Open EPI calculator at 95% confidence interval was used which yielded a sample of 384. This sample size is calculated on the assumption that 50% of participants possess some knowledge of osteoporosis (6). However, for statistical convenience we recruited 400 subjects.**

***6. Lulla D, Teo CW, Shen X, Loi ZB, Quek KW, Lis HL, Koh SA, Chan ET, Lim SW, Low LL. Assessing the knowledge, attitude and practice of osteoporosis among Singaporean women aged 65 years and above at two SingHealth polyclinics. Singapore Medical Journal. 2021 Apr 1;62(4):190-4.***

Data management is extremely important for research using social media, as in this study. Please describe in detail how you maintained the quality of the data.
**Response:**

**To ensure the quality of the data, we ensured that the following steps were undertaken.**

- **Research question and survey question was well-defined prior to the collection of the data.**
- **Easily comprehensible OPAAT tool was used. The other surveys in which this tool was used reported good understanding of the questions from the participants’ end.**
- **The forms were disseminated ethically and transparently through social media where our target population was likely to be found, ensuring a variety of respondents.**
- **The data was fully checked and assessed for inconsistencies, errors and incompletion. If found, this missing data was supposed to be removed from the data set, which did not happen in our case as all the responses were complete.**
- **Data was protected and available only to the research team, primarily the principal investigator and statistician.**
- **Appropriate data analysis techniques were used to answer the research question.**

Results
Line 78 What was the response rate? Does it mean it was 100%? Please state.
**Response: Yes, the response rate was 100%. This is mentioned in the first line of the Results section.**

Discussion
Please provide more details on strength and limitation, such as how limitation may not apply to the general public. In terms of limitation, since the study participants were only social media users, the findings cannot be generalized to the general population.

Please describe what proposed solution is possible based on the results of this study. It would be a very useful paper if specific solutions could be mentioned that would raise the level of knowledge of osteoporosis among the people of Pakistan.

**Response: These are valid points and have been addressed in the manuscript. Thank you for your valuable review.**
